# Crystal structure and Hirshfeld surface analysis of 1-(2-fluoro­phen­yl)-1*H*-tetra­zole-5(4*H*)-thione

**DOI:** 10.1107/S2056989020007033

**Published:** 2020-06-05

**Authors:** Rizvan K. Askerov, Abel M. Maharramov, Ali N. Khalilov, Mehmet Akkurt, Anzurat A. Akobirshoeva, V. K. Osmanov, A. V. Borisov

**Affiliations:** a Organic Chemistry Department, Baku State University, Z. Xalilov str. 23, Az, 1148 Baku, Azerbaijan; b Department of Physics and Chemistry, "Composite Materials" Scientific Research Center, Azerbaijan State Economic University (UNEC), H. Aliyev str. 135, Az 1063, Baku, Azerbaijan; c Department of Physics, Faculty of Sciences, Erciyes University, 38039 Kayseri, Turkey; d Acad. Sci. Republ. Tadzhikistan, Kh. Yu. Yusufbekov Pamir Biol. Inst., 1 Kholdorova St, Khorog 736002, Gbao, Tajikistan; e Nizhny Novgorod State Technical University n.a. R.E. Alekseev, Nizhny Novgorod, Russian Federation

**Keywords:** crystal structure, hydrogen bonding, cyclo­addition products, 1*H*-tetra­zole-5-thione ring, π–π stacking inter­actions

## Abstract

In the crystal of the title compound, C_7_H_5_FN_4_S, the mol­ecules are non-planar, with dihedral angle formed by least-squares planes of tetra­zole and benzene rings of 59.94 (8) °. The crystal packing is formed by N—H⋯S hydrogen bonds, which link the mol­ecules into centrosymmetric dimers with an *R* ^2^_2_(8) ring motif, and by the offset face-to-face π–π stacking inter­actions between the benzene rings, which join the dimers into layers parallel to (100). The Hirshfeld surface analysis shows that the most important contributions to the surface contacts are from N⋯H/H⋯N (21.9%), S⋯H/H⋯S (21.1%), H⋯H (14.6%), F⋯H/H⋯F (11.8%) and C⋯H/H⋯C (9.5%) inter­actions.

## Abstract 2

In the crystal, mol­ecules are linked by pairs of N—H⋯S hydrogen bonds, forming inversion dimers with an 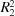
(8) ring motif. The dimers are linked by the offset face-to-face π–π stacking inter­actions.

## Chemical context   

Tetra­zoles as an important class of five-membered heterocyclic compounds have been known for over a hundred years. The most common synthetic approach to construct tetra­zoles, based on the reaction of nitriles with hydrazoic acid, was first discovered by Hantzsch & Vagt (1901 ▸). Up to know, most synthetic protocols comprise the cyclo­addition of nitriles, thio­cyanates or iso­thio­cyanates with an azide moiety, under different conditions. Tetra­zole derivatives have found a broad range of applications in medicinal chemistry (Wang *et al.*, 2019 ▸; Gao *et al.*, 2019 ▸; Arulmozhi *et al.*, 2017 ▸), coordination chemistry (Askerov *et al.*, 2018 ▸; Askerov *et al.*, 2019*a* ▸,*b* ▸; Aromí *et al.*, 2011 ▸) and material science (Frija *et al.*, 2010 ▸; Lv *et al.*, 2006 ▸). Numerous tetra­zole-based synthetic compounds such as tomelukast, cefazolin, losartan, valsartan and alfentanil have already been used in medicinal practice.
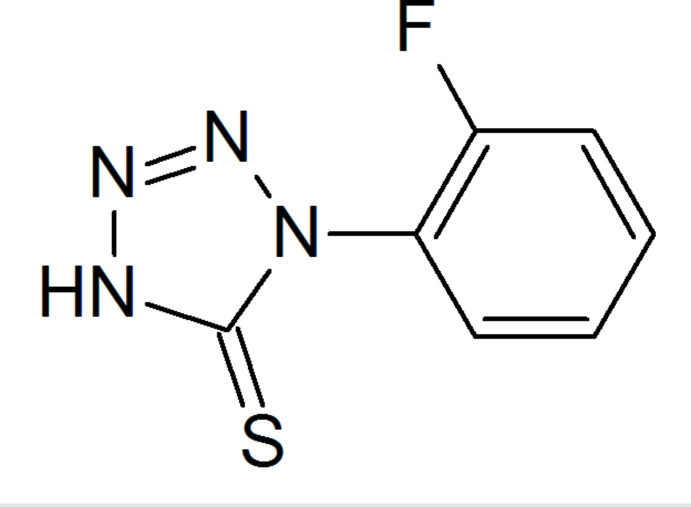


As a result of the considerable inter­est in this field, significant developments in the synthesis of tetra­zoles have been attained, which were recently reviewed (Neochoritis *et al.*, 2019 ▸). As a further study of the chemistry of tetra­zoles, herein we report the crystal structure and Hirshfeld surface analysis of the title compound.

## Structural commentary   

**Figure 1:**
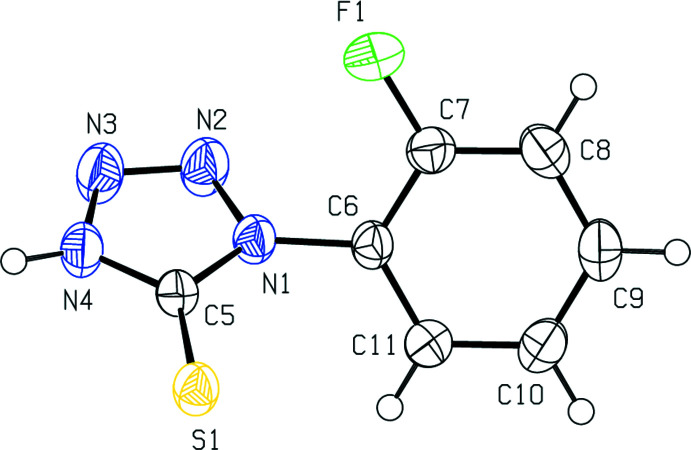
The mol­ecular structure of the title compound, showing displacement ellipsoids drawn at the 50% probability level.

The mol­ecule of the title compound (Fig. 1 ▸) is non-planar. The five-membered 4-di­hydro-5*H*-tetra­zole ring (N1–N4/C5) is essentially planar, with a largest deviation of 0.005 (1) Å for N3. The dihedral angle between the mean planes of the tetra­zole and benzene rings is 59.94 (8)°. The bond dimensions are typical of similar compounds, with a distinct N2=N3 double bond.

## Supra­molecular features   

**Figure 2:**
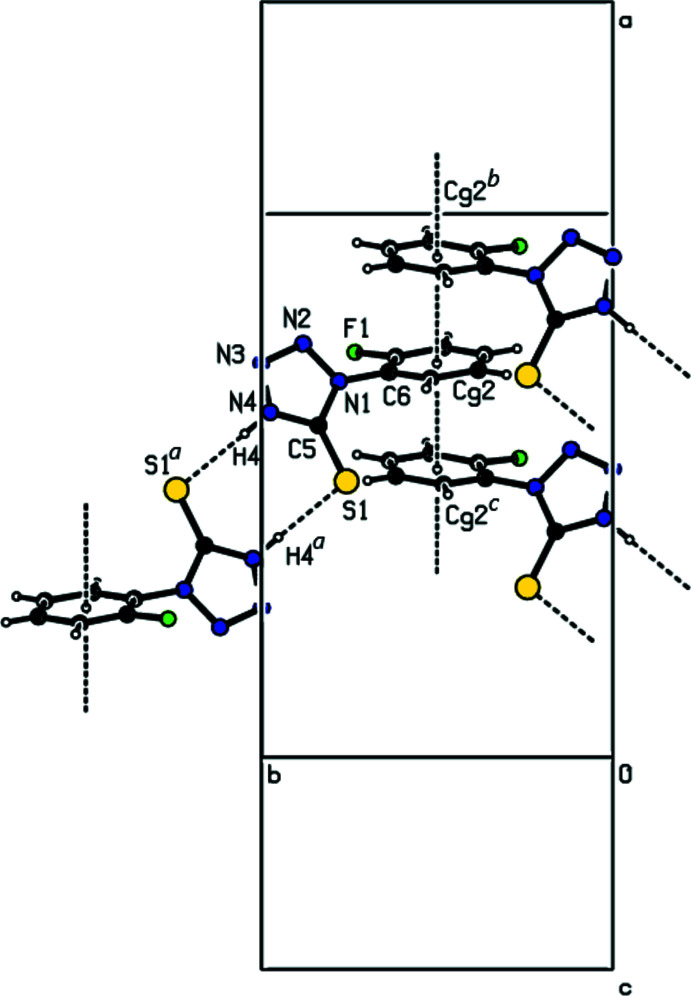
Crystal packing of the title compound viewed along the *a*-axis direction. Dashed lines indicate the N—H⋯S hydrogen bonds, which form centrosymmetric dimers with an 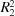
(8) ring motif, and the face-to-face π–π stacking inter­actions, which connect the dimers into layers parallel to (100).

In the crystal, mol­ecules are linked by pairs of N—H⋯S hydrogen bonds, forming centrosymmetric dimers with an 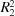
(8) ring motif (see Fig. 2 ▸ and Table 1 ▸). The dimers are linked by the offset face-to-face π–π stacking inter­actions between the benzene rings, which are characterized by inter­centroid distances of 3.8963 (9) and 3.8964 (9) Å, and centroid-to-plane distances of 3.4589 (6) and 3.4578 (6) Å (Fig. 2 ▸). Neighbouring mol­ecules within the stack are related by the *c* glide plane. The hydrogen bonds and stacking inter­actions link the mol­ecules into layers parallel to (100). Other short inter­molecular contacts are collected in Table 2 ▸.

## Hirshfeld surface analysis   

**Figure 3:**
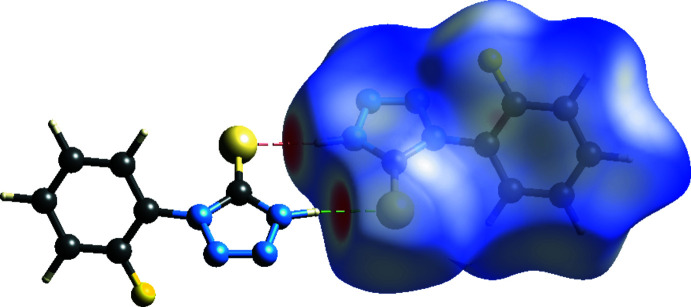
A view of the three-dimensional Hirshfeld surface for the title mol­ecule, plotted over *d*_norm_ ranging from −0.4612 to 1.2843 a.u. A dimer formed by N—H⋯S hydrogen bonds is shown.

**Figure 4:**
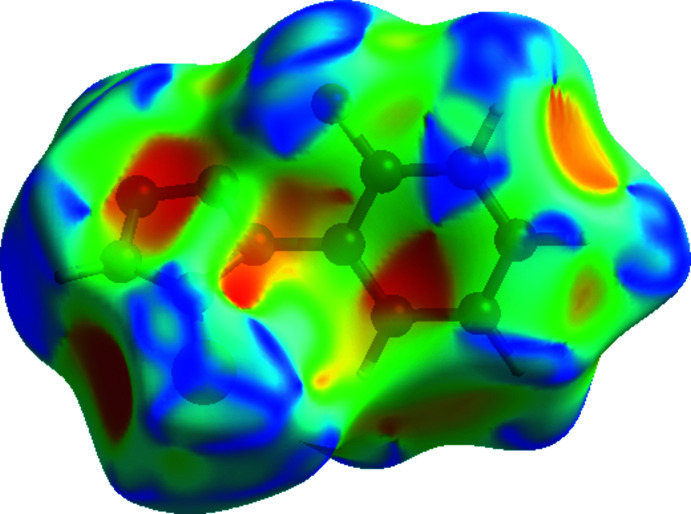
Hirshfeld surface of the title mol­ecule plotted over shape-index.

**Figure 5:**
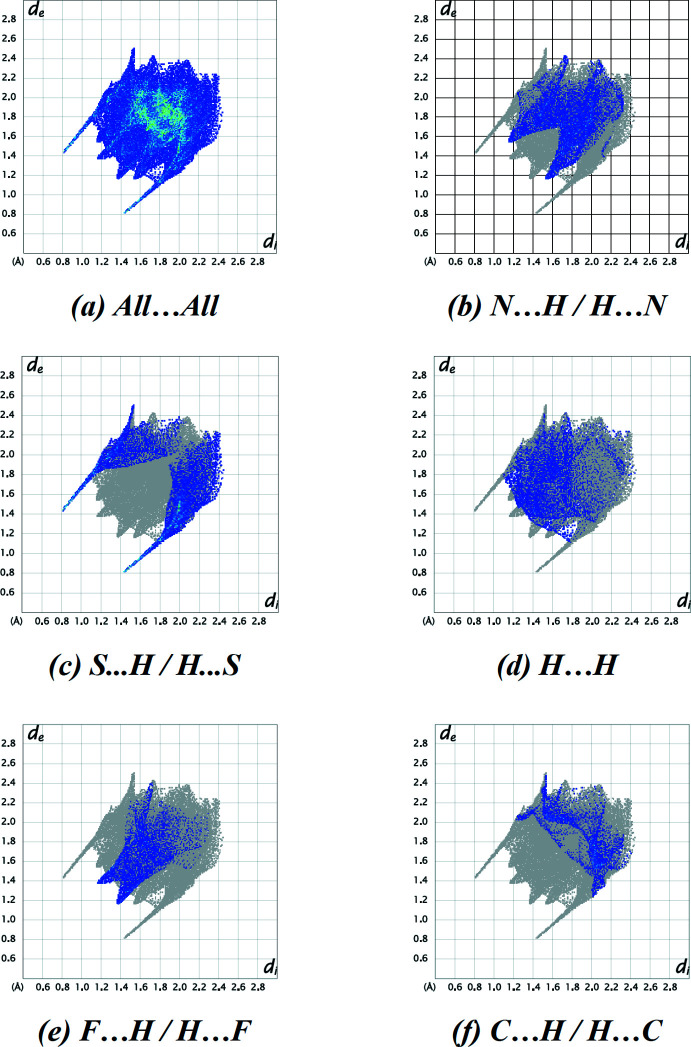
A view of two-dimensional fingerprint plots for the title compound, showing (*a*) all inter­actions, and delineated into (*b*) N⋯H/H⋯N, (*c*) S⋯H/H⋯S, (*d*) H⋯H, (*e*) F⋯H/H⋯F and (*f*) C⋯H/H⋯C inter­actions. The *d*_i_ and *d*_e_ values are the closest inter­nal and external distances (in Å) from given points on the Hirshfeld surface contacts.

In order to investigate the inter­molecular inter­actions in the crystal structure of the title compound in a visual manner, Hirshfeld surfaces (McKinnon *et al.*, 2007 ▸) and their associated two-dimensional fingerprint plots (Spackman & McKinnon, 2002 ▸) were generated using *CrystalExplorer17* (Turner *et al.*, 2017 ▸). The shorter and longer contacts are indicated as red and blue spots, respectively, on the Hirshfeld surfaces, and contacts with distances approximately equal to the sum of the van der Waals radii are represented as white spots. The contribution of inter­atomic contacts (Table 2 ▸) to the *d*_norm_ surface of the title compound is shown in Fig. 3 ▸. In Fig. 4 ▸, red and blue triangles can be seen on the shape-index surface, which indicate the presence of π–π stacking inter­actions in the crystal structure. Analysis of the two-dimensional fingerprint plots (Fig. 5 ▸) reveals that N⋯H/H⋯N (21.9%) and S⋯H/H⋯S (21.1%) contacts (*i.e.* N—H⋯S) are the major contributors to the Hirshfeld surface, while H⋯H (14.6%), F⋯H/H⋯F (11.8%) and C⋯H/H⋯C (9.5%) contacts make a less significant contribution. The contribution of the C⋯C (6.6%) (*i.e.* π–π stacking) contacts and other contacts such as N⋯N (2.8%), F⋯C/C⋯F (2.4%), N⋯C/C⋯N (2.4%), F⋯N/N⋯F (1.7%), S⋯N/N⋯S (1.7%), S⋯C/C⋯S (1.7%), F⋯F (1.5%) and S⋯S (0.4%) make a small contribution to the overall Hirshfeld surface.

## Database survey   

A search of the Cambridge Crystallographic Database (CSD version 5.40, update of September 2019; Groom *et al.*, 2016 ▸) yielded nine entries closely related to the title compound, *viz*. 1-(4-fluoro­phen­yl)-4,4,6-trimethyl-3,4-di­hydro­pyrimidine-2(1*H*)-thione (CSD refcode ASEHIR; Kadir *et al.*, 2016 ▸), 3-(adamantan-1-yl)-4-(4-fluoro­phen­yl)-1-[(4-phenyl­piperazin-1-yl)meth­yl]-4,5-di­hydro-1*H*-1,2,4-triazole-5-thione (ZEFKED; Al-Alshaikh *et al.*, 2017 ▸), 3-(adamantan-1-yl)-4-(4-fluoro­phen­yl)-1-{[4-(2-meth­oxy­phen­yl)piperazin-1-yl]-meth­yl}-4,5-di­hydro-1*H*-1,2,4-triazole-5-thione (ZEFKAZ; Al-Alshaikh *et al.*, 2017 ▸), 3-(adamantan-1-yl)-4-(2-bromo-4-fluoro­phen­yl)-1*H*-1,2,4-triazole-5(4*H*)-thione (ZOZNEK; Abdelrazeq *et al.*, 2020 ▸), 2-fluoro-*N*-(3-(methyl­sulfan­yl)-1*H*-1,2,4-triazol-5-yl)benzamide (MITMOU; Moreno-Fuquen *et al.*, 2019 ▸), (5-amino-3-(methyl­sulfan­yl)-1*H*-1,2,4-triazol-1-yl)(2-fluoro­phen­yl)methanone (MITMIO; Moreno-Fuquen *et al.*, 2019 ▸), 4-(benzo[*b*]thio­phen-2-yl)-5-(3,4,5-tri­meth­oxy­phen­yl)-2*H*-1,2,3-triazole (PONWIA; Penthala *et al.*, 2014 ▸), 4-(benzo[*b*]thiophen-2-yl)-2-methyl-5-(3,4,5-tri­meth­oxy­phen­yl)-2*H*-1,2,3-triazole (PONWOG; Penthala *et al.*, 2014 ▸), (*E*)-3-(4-fluoro­phen­yl)-1-[1-(4-fluoro­phen­yl)-5-methyl-1*H*-1,2,3-triazol-4-yl]prop-2-en-1-one (MESTAI; El-Hiti *et al.*, 2018 ▸), 4-amino-3-methyl-5-(*p*-tol­yl)-4*H*-1,2,4-triazole (JESTOR; Şahin *et al.*, 2006 ▸), 4-amino-3-methyl-5-phenyl-4*H*-1,2,4-triazole (JESTUX; Şahin *et al.*, 2006 ▸), and 2-phenyl-4,5-dianilino-2*H*-1,2,3-triazole (PANTZL10; Harlow *et al.*, 1977 ▸).

In the crystal of ASEHIR, pairs of mol­ecules related by the twofold rotation axis are linked by N—H⋯S hydrogen bonds, forming dimers.

The crystal structure of ZEFKED shows pairs of C—H⋯F hydrogen bonds forming inversion dimers, while in the crystal of ZEFKAZ, in addition to the C—H⋯F hydrogen bonds that generate chains parallel to the *b* axis, there are C—H⋯π inter­actions that link the chains to form layers parallel to the *ab* plane.

In the crystal of ZOZNEK, the mol­ecules are linked by weak C—H⋯π(phen­yl) inter­actions, forming supra­molecular chains extending along the *c-*axis direction. The crystal packing is further consolidated by inter­molecular N—H⋯S hydrogen bonds and by weak C—H⋯S inter­actions, yielding double chains propagating along the *a*-axis direction.

In the crystal structure of MITMOU, the supra­molecular assembly is formed mainly by (N,C)—H⋯(N,O) hydrogen-bond inter­actions. Initially, strong N—H⋯N hydrogen bonds link pairs of inversion-related mol­ecules that act as slabs of infinite chains running along the [100] direction connected by a C—H⋯O hydrogen bond. Along the [010] direction, neighbouring chains are further connected by weak π–π inter­actions between two arene rings of adjacent mol­ecules.

The crystal structure of MITMIO is built by a combination of strong N—H⋯O and N—H⋯N hydrogen bonds, which form chains of mol­ecules running along the [100] direction. Parallel inversion-related chains of mol­ecules are further connected by weaker C—H⋯O inter­actions to build the mol­ecular architecture along the [001] direction. Weak C—H⋯N inter­actions connect the mol­ecules in order to complete the three-dimensional structure along the [010] direction.

In the crystal of PONWIA, the mol­ecules are linked into chains by N—H⋯O hydrogen bonds with 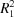
(5) ring motifs. After the *N*-methyl­ation of the PONWIA mol­ecule, no hydrogen-bonding inter­actions were observed for structure PONWOG. The crystal structure of PONWOG shows a disorder due to a 180° flip of the benzo­thio­phene ring system.

In the crystal of MESTAI, the asymmetric unit comprises two mol­ecules with similar conformations. In the crystal, weak C—H⋯F inter­actions form chains of mol­ecules and the chains are stacked to form layers parallel to (101).

In JESTOR, mol­ecules are linked principally by N—H⋯N hydrogen bonds involving the amino NH_2_ group and a triazole N atom, forming 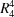
(20) and 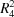
(10) rings that combine to give a three-dimensional network of mol­ecules. The hydrogen bonding is supported by two different C—H⋯π inter­actions from the tolyl ring to either a triazole ring or a tolyl ring in a neighboring mol­ecule. In JESTUX, inter­molecular hydrogen bonds and C—H⋯π inter­actions generate 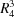
(15) and 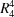
(21) rings.

## Synthesis and crystallization   

To a solution of of NaN_3_ (29 mmol) in 50 mL of H_2_O 2-fluoro­phenyl­iso­thio­cyanate (19.6 mmol) was added at 293 K. The reaction mixture was boiled for 2 h, cooled to 293 K; then the aqueous solution was filtered from undissolved impurities and a 10% aqueous solution of HCl was added to it with stirring to pH = 2. The precipitate of the title compound was filtered off, washed with water, and then the product was recrystallized from ethanol.

1-(2-Fluoro­phen­yl)-1*H*-tetra­zole-5(4*H*)-thione: yield 72% as white powder, m.p. 426 K. Analysis calculated for C_7_H_5_FN_4_S (%): C 42.85, H 2.57, N 28.56. Found (%): C 42.62, H 2.66, N 28.59. ^1^H NMR (400.00 MHz, DMSO-*d*_6_): *δ* = 7.73 (*m*, 1H), 7.68 (*m*, 1H), 7.55 (*t*, 1H), 7.45 (*t*, 1H). ^13^C NMR (100.60 MHz, DMSO-*d*_6_): *δ* = 162.76 (**C**=S), 156.39 (**C**—F), [137.33 (**C**—N, Ar), 128.76, 127.08, 124.94, 114.88 (4**C**H, Ar)].

## Refinement details   

Crystal data, data collection and structure refinement details are summarized in Table 3 ▸. The C-bound H atoms were placed in calculated positions (0.93 Å) and refined as riding with *U*_iso_(H) = 1.2*U*_eq_(C). The N-bound H atom was located in a difference map and refined isotropically.

## Supplementary Material

Crystal structure: contains datablock(s) I. DOI: 10.1107/S2056989020007033/yk2131sup1.cif

Structure factors: contains datablock(s) I. DOI: 10.1107/S2056989020007033/yk2131Isup2.hkl

Supporting information file. DOI: 10.1107/S2056989020007033/yk2131Isup3.cml

CCDC reference: 2005595

Additional supporting information:  crystallographic information; 3D view; checkCIF report

## Figures and Tables

**Table 1:** Hydrogen-bond geometry (Å, °)

*D*—H⋯*A*	*D*—H	H⋯*A*	*D*⋯*A*	*D*—H⋯*A*
N4—H4⋯S1^i^	0.90 (2)	2.35 (2)	3.2456 (12)	173.2 (18)

Symmetry code: (i) 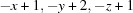.

**Table 2:** Summary of short inter­atomic contacts (Å) in the title compound

Contact	Distance	Symmetry operation
S1⋯S1	3.7741 (6)	1 − *x*, *y*, 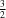 − *z*
C5⋯S1	3.6367 (13)	1 − *x*, *y*, 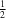 − *z*
H4⋯S1	2.35	1 − *x*, 2 − *y*, 1 − *z*
H10*A*⋯S1	3.18	1 − *x*, 1 − *y*, 1 − *z*
S1⋯H10*A*	3.04	*x*, 1 − *y*, 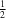 + *z*
F1⋯H8*A*	2.63	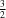 − *x*, 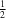 + *y*, 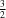 − *z*
F1⋯F1	3.0330 (15)	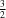 − *x*, 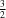 − *y*, 1 − *z*
N3⋯H10*A*	2.82	*x*, 1 + *y*, *z*
N3⋯C5	3.38	*x*, 2 − *y*, − 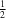 + *z*

**Table 3:** Experimental details

Crystal data	
Chemical formula	C_7_H_5_FN_4_S
*M*_r_	196.21
Crystal system, space group	Monoclinic, *C*2/*c*
Temperature (K)	296
*a*, *b*, *c* (Å)	23.5593 (11), 9.2849 (5), 7.7927 (4)
β (°)	104.009 (1)
*V* (Å^3^)	1653.92 (15)
*Z*	8
Radiation type	Mo *K*α
μ (mm^−1^)	0.36
Crystal size (mm)	0.23 × 0.15 × 0.08

Data collection	
Diffractometer	Bruker APEXII CCD
Absorption correction	Multi-scan (*SADABS*; Bruker, 2003 ▸)
*T*_min_, *T*_max_	0.690, 0.746
No. of measured, independent and observed [*I* > 2σ(*I*)] reflections	9005, 2406, 2135
*R*_int_	0.017
(sin θ/λ)_max_ (Å^−1^)	0.713

Refinement	
*R*[*F* ^2^ > 2σ(*F* ^2^)], *wR*(*F* ^2^), *S*	0.036, 0.098, 1.00
No. of reflections	2406
No. of parameters	122
H-atom treatment	H atoms treated by a mixture of independent and constrained refinement
Δρ_max_, Δρ_min_ (e Å^−3^)	0.29, −0.22

Computer programs: *APEX2* and *SAINT* (Bruker, 2003 ▸), *SHELXT2014/5* (Sheldrick, 2015*a* ▸), *SHELXL2018/3* (Sheldrick, 2015*b* ▸), *ORTEP-3 for Windows* (Farrugia, 2012 ▸) and *PLATON* (Spek, 2020 ▸).

## supplementary crystallographic information

### Crystal data

**Table:**

C_7_H_5_FN_4_S	*F*(000) = 800
*M**_r_* = 196.21	*D*_x_ = 1.576 Mg m^−^^3^
Monoclinic, *C*2/*c*	Mo *K*α radiation, λ = 0.71073 Å
*a* = 23.5593 (11) Å	Cell parameters from 4417 reflections
*b* = 9.2849 (5) Å	θ = 2.4–30.5°
*c* = 7.7927 (4) Å	µ = 0.36 mm^−^^1^
β = 104.009 (1)°	*T* = 296 K
*V* = 1653.92 (15) Å^3^	Prism, colourless
*Z* = 8	0.23 × 0.15 × 0.08 mm

### Data collection

**Table:**

Bruker APEXII CCD diffractometer	2135 reflections with *I* > 2σ(*I*)
φ and ω scans	*R*_int_ = 0.017
Absorption correction: multi-scan (SADABS; Bruker, 2003)	θ_max_ = 30.5°, θ_min_ = 1.8°
*T*_min_ = 0.690, *T*_max_ = 0.746	*h* = −32→33
9005 measured reflections	*k* = −13→13
2406 independent reflections	*l* = −10→10

### Refinement

**Table:**

Refinement on *F*^2^	Primary atom site location: difference Fourier map
Least-squares matrix: full	Secondary atom site location: difference Fourier map
*R*[*F*^2^ > 2σ(*F*^2^)] = 0.036	Hydrogen site location: mixed
*wR*(*F*^2^) = 0.098	H atoms treated by a mixture of independent and constrained refinement
*S* = 1.00	*w* = 1/[σ^2^(*F*_o_^2^) + (0.0487*P*)^2^ + 1.1614*P*] where *P* = (*F*_o_^2^ + 2*F*_c_^2^)/3
2406 reflections	(Δ/σ)_max_ = 0.001
122 parameters	Δρ_max_ = 0.29 e Å^−^^3^
0 restraints	Δρ_min_ = −0.22 e Å^−^^3^

### Special details

**Table:**

Geometry. All esds (except the esd in the dihedral angle between two l.s. planes) are estimated using the full covariance matrix. The cell esds are taken into account individually in the estimation of esds in distances, angles and torsion angles; correlations between esds in cell parameters are only used when they are defined by crystal symmetry. An approximate (isotropic) treatment of cell esds is used for estimating esds involving l.s. planes.

### Fractional atomic coordinates and isotropic or equivalent isotropic displacement parameters (Å^2^)

**Table:**

	*x*	*y*	*z*	*U*_iso_*/*U*_eq_	
S1	0.51038 (2)	0.75776 (4)	0.51736 (5)	0.03900 (12)	
F1	0.71634 (4)	0.73413 (10)	0.64199 (14)	0.0536 (3)	
N1	0.61196 (5)	0.77955 (12)	0.40738 (16)	0.0346 (2)	
N2	0.64339 (6)	0.88176 (13)	0.3424 (2)	0.0480 (3)	
N3	0.61568 (6)	1.00050 (13)	0.3346 (2)	0.0494 (3)	
N4	0.56649 (5)	0.97577 (12)	0.39123 (17)	0.0399 (3)	
H4	0.5428 (9)	1.048 (2)	0.408 (3)	0.060 (5)*	
C5	0.56255 (5)	0.83768 (13)	0.43970 (17)	0.0320 (2)	
C6	0.63424 (5)	0.63719 (13)	0.44289 (17)	0.0314 (2)	
C7	0.68772 (6)	0.61740 (14)	0.56178 (18)	0.0361 (3)	
C8	0.71115 (6)	0.48238 (16)	0.6014 (2)	0.0434 (3)	
H8A	0.747433	0.470495	0.680048	0.052*	
C9	0.67955 (7)	0.36465 (16)	0.5217 (2)	0.0444 (3)	
H9A	0.694563	0.272435	0.547932	0.053*	
C10	0.62584 (7)	0.38250 (15)	0.4034 (2)	0.0419 (3)	
H10A	0.604979	0.302279	0.351172	0.050*	
C11	0.60297 (6)	0.51874 (14)	0.36219 (19)	0.0369 (3)	
H11A	0.567110	0.530830	0.281472	0.044*	

### Atomic displacement parameters (Å^2^)

**Table:**

	*U*^11^	*U*^22^	*U*^33^	*U*^12^	*U*^13^	*U*^23^
S1	0.03642 (19)	0.03299 (18)	0.0519 (2)	0.00355 (12)	0.01903 (15)	0.00288 (13)
F1	0.0467 (5)	0.0476 (5)	0.0603 (6)	−0.0097 (4)	0.0012 (4)	−0.0104 (4)
N1	0.0316 (5)	0.0285 (5)	0.0465 (6)	0.0026 (4)	0.0148 (4)	0.0020 (4)
N2	0.0431 (6)	0.0347 (6)	0.0728 (9)	0.0013 (5)	0.0267 (6)	0.0084 (6)
N3	0.0476 (7)	0.0330 (6)	0.0737 (9)	0.0031 (5)	0.0266 (6)	0.0085 (6)
N4	0.0404 (6)	0.0291 (5)	0.0529 (7)	0.0056 (4)	0.0165 (5)	0.0035 (5)
C5	0.0314 (5)	0.0287 (6)	0.0356 (6)	0.0034 (4)	0.0076 (5)	−0.0012 (4)
C6	0.0311 (5)	0.0277 (5)	0.0378 (6)	0.0035 (4)	0.0126 (5)	0.0009 (4)
C7	0.0334 (6)	0.0351 (6)	0.0400 (6)	−0.0017 (5)	0.0095 (5)	−0.0028 (5)
C8	0.0350 (6)	0.0463 (8)	0.0475 (8)	0.0082 (6)	0.0072 (6)	0.0077 (6)
C9	0.0486 (8)	0.0338 (7)	0.0548 (8)	0.0104 (6)	0.0202 (6)	0.0088 (6)
C10	0.0472 (7)	0.0307 (6)	0.0508 (8)	−0.0028 (5)	0.0178 (6)	−0.0032 (6)
C11	0.0345 (6)	0.0338 (6)	0.0421 (7)	−0.0007 (5)	0.0086 (5)	−0.0014 (5)

### Geometric parameters (Å, º)

**Table:**

S1—C5	1.6696 (13)	C6—C11	1.3868 (18)
F1—C7	1.3476 (15)	C7—C8	1.3745 (19)
N1—C5	1.3608 (15)	C8—C9	1.382 (2)
N1—N2	1.3735 (16)	C8—H8A	0.9300
N1—C6	1.4244 (16)	C9—C10	1.384 (2)
N2—N3	1.2752 (16)	C9—H9A	0.9300
N3—N4	1.3558 (17)	C10—C11	1.3818 (19)
N4—C5	1.3462 (17)	C10—H10A	0.9300
N4—H4	0.90 (2)	C11—H11A	0.9300
C6—C7	1.3836 (18)		
			
C5—N1—N2	110.84 (11)	F1—C7—C6	118.43 (12)
C5—N1—C6	128.58 (11)	C8—C7—C6	121.51 (12)
N2—N1—C6	120.44 (10)	C7—C8—C9	118.49 (13)
N3—N2—N1	107.44 (11)	C7—C8—H8A	120.8
N2—N3—N4	107.81 (11)	C9—C8—H8A	120.8
C5—N4—N3	112.04 (11)	C8—C9—C10	120.72 (13)
C5—N4—H4	125.3 (13)	C8—C9—H9A	119.6
N3—N4—H4	122.1 (13)	C10—C9—H9A	119.6
N4—C5—N1	101.86 (11)	C11—C10—C9	120.44 (13)
N4—C5—S1	129.19 (10)	C11—C10—H10A	119.8
N1—C5—S1	128.94 (10)	C9—C10—H10A	119.8
C7—C6—C11	119.73 (12)	C10—C11—C6	119.10 (13)
C7—C6—N1	119.10 (11)	C10—C11—H11A	120.4
C11—C6—N1	121.17 (11)	C6—C11—H11A	120.4
F1—C7—C8	120.05 (12)		
			
C5—N1—N2—N3	0.45 (18)	N2—N1—C6—C11	−122.21 (15)
C6—N1—N2—N3	−175.58 (13)	C11—C6—C7—F1	−178.12 (12)
N1—N2—N3—N4	−0.81 (18)	N1—C6—C7—F1	1.09 (18)
N2—N3—N4—C5	0.94 (19)	C11—C6—C7—C8	0.5 (2)
N3—N4—C5—N1	−0.62 (16)	N1—C6—C7—C8	179.75 (13)
N3—N4—C5—S1	179.93 (11)	F1—C7—C8—C9	177.56 (13)
N2—N1—C5—N4	0.11 (15)	C6—C7—C8—C9	−1.1 (2)
C6—N1—C5—N4	175.73 (13)	C7—C8—C9—C10	0.7 (2)
N2—N1—C5—S1	179.57 (11)	C8—C9—C10—C11	0.3 (2)
C6—N1—C5—S1	−4.8 (2)	C9—C10—C11—C6	−0.9 (2)
C5—N1—C6—C7	−116.67 (15)	C7—C6—C11—C10	0.4 (2)
N2—N1—C6—C7	58.59 (18)	N1—C6—C11—C10	−178.76 (12)
C5—N1—C6—C11	62.53 (19)		

### Hydrogen-bond geometry (Å, º)

**Table:**

*D*—H···*A*	*D*—H	H···*A*	*D*···*A*	*D*—H···*A*
N4—H4···S1^i^	0.90 (2)	2.35 (2)	3.2456 (12)	173.2 (18)
C5—S1···*Cg*1^ii^	1.67 (1)	3.77 (1)	4.0760 (14)	88 (1)

Symmetry codes: (i) −*x*+1, −*y*+2, −*z*+1; (ii) −*x*+1, *y*, −*z*+1/2.
